# Letter to editor: is laboratory index really a practical and valid tool to predict mortality?

**DOI:** 10.1186/s12877-021-02478-2

**Published:** 2021-10-09

**Authors:** Merve Güner Oytun, Polat Ercan, Serdar Ceylan, Arzu Okyar Baş, Meltem Halil, Mustafa Cankurtaran, Burcu Balam Doğu

**Affiliations:** 1 Faculty of Medicine, Department of Internal Medicine, Division of Geriatrics, Hacettepe University, Sıhhıye, 06410 Ankara, Turkey; 2 Faculty of Medicine, Department of Internal Medicine, Hacettepe University, Ankara, Turkey

**Keywords:** Mortality Index, Laboratory Index, Frailty

## Abstract

We carefully studied the article titled “A practical laboratory index to predict institutionalization and mortality – an 18-year population-based follow-up study” written by Heikkilä et al. and published in BMC Geriatrics on 25 February 2021 with great interest. We would like to make some comments regarding this article and tool. Laboratory Index (LI) has been executed with the data of 728 patients who had followed-up in our center, however the LI score was not able to predict the 10-year and 18-year mortality. Therefore, a question mark has been aroused in our minds at some points. Neither frailty nor comorbidities were considered in this index. For a geriatric patient, it would be inadequate to evaluate laboratory results regardless of the clinical status. Similarly, it would not be appropriate to predict mortality only on the basis of laboratory results without considering the clinical status of the patient.

We think that although the recent study has a great impact, it can be improved by incorporating data on the comorbidities and frailty status of the patients into the analysis.

Dear Editor,

We carefully studied the article titled “A practical laboratory index to predict institutionalization and mortality – an 18-year population-based follow-up study” written by Heikkilä et al. and published in BMC Geriatrics on 25 February 2021 with great interest. The large sample size and the length of follow-up duration were the striking parts of the paper. Additionally, the investigated tool seems very useful and easy to apply. To determine a practical and useful tool to predict the risk of mortality and institutionalization is of great importance for geriatric patients. From this point of view, this study adds important knowledge to the literature. However, when we implemented this tool to our patients’ data, the predictions were not compatible with the exact mortality rates. Therefore, we would like to make some comments regarding this article and prediction tool.

Laboratory index (LI) has been executed for our followed-up 728 patients, with the median age 70.0 ranged between 65 and 92 years old. The vital status of 728 patients and the date of the death who were deceased were determined through linkage with the Turkish national death registry. In the 10-year follow-up, 24 % of all patients were dead, and 60.2 % of the 728 patients died in an 18-year follow-up. Index score 0.09 or over and 0.15 or over did not predict the mortality during 10-year follow-up (HR:1.08 %95 CI 0.70–1.65 and HR: 1.31 %95 CI 0.86-2.00, respectively and p values are > 0.05). Index score 0.09 or over also did not predict mortality for the 18-year follow-up (HR: 1.19 95 % CI 0.91–1.54, p value > 0.05). These associations were still not significant after age and gender adjustments. Therefore, a question mark has been aroused in our minds at some points.

In the study of Heikkilä et al., authors had created a laboratory index that included fourteen entities including albumin, creatinine, thyroid-stimulating hormone (TSH), C-reactive protein (CRP), and the index had been constructed according to the value of subjects off the normal limits [1]. Traditionally, reference limits are based on young and middle-aged adults. However, for the geriatric age group, reference values of certain laboratory tests might differ from the younger adults, as many physiological changes occur during the aging process. Besides, some temporary or progressive changes in laboratory parameters may occur for older adults. For example, albumin levels decrease in malnutrition, frailty, and also in acute illness states [2]. There is a dynamic change in serum albumin levels in the state of acute illness which is an indicator of intra-individual variability. On the other hand, serum cholesterol level is one of the markers of cardiovascular risk, cholesterol levels begin to decrease starting from the sixth decade of life [3]. Higher reference intervals for TSH levels should be accepted as normal since it is known that age-specific increases in population TSH distribution occur [4]. Furthermore, renal markers such as urea, and uric acid all tend to increase with age, as glomerular filtration rate (GFR) decreases [5]. Serum creatinine levels may be lower than younger ones because of the age-related decrease in muscle mass. Even though serum creatinine levels are within normal ranges, kidney functions could be diminished [6]. The difference between creatinine and GFR is more prominent in sarcopenic patients. These show the importance of the clinical profile of the patient. Whether the patient has frailty or not, sarcopenia or not, inflammatory conditions or not, cardiovascular diseases or not have an influence on laboratory parameters. Therefore, a holistic approach is necessary for assessing a geriatric patient. To make predictions by taking only laboratory parameters into account seems inadequate. Normal ranges of geriatric patients also differ from the ones of younger adults. So, there is this question, how far the conception of reference intervals, which is one of the most important fundamentals in laboratory medicine, is still valid for geriatric patients?

Another troubling part of the current paper is that mortality and institutionalization rates were not adjusted for multi-morbidity. It is well-known that most of the older adults had two or more disorders [7]. And the risk of mortality increases as the number of the comorbidities increase, according to studies by Menotti et al., Deeg et al., and Byles et al. [8]. In the present study, data related to the comorbidity status of the study population were not mentioned and their probable effect on mortality was not analyzed. As mentioned by the authors, LI did not predict the institutionalization due to not including the frailty index. Comprehensive geriatric assessment might have helped to determine the frailty status of the study population. Neither frailty nor comorbidity were considered in this index. For a geriatric patient, it would be inadequate to evaluate laboratory results regardless of the clinical status. Similarly, it would not be appropriate to predict mortality only on the basis of laboratory results without considering the clinical status of the patient.

We think that although the recent study has a great impact, it can be improved by incorporating data on the comorbidities and frailty status of the patients into the analysis. Future studies should focus on a more comprehensive but still practical and easy-to-apply tool to predict mortality and institutionalization risk of geriatric patients.

## Data Availability

The data that support the findings of this study are available from the corresponding author, M.G.O., upon reasonable request.
